# Temporomandibular Disorder Treatment With Diathermy Stimulation Technique: A Pilot Study

**DOI:** 10.1155/ijod/5486917

**Published:** 2025-12-08

**Authors:** Funda Goker, Andrea Gizdulich, Ishita Singhal, Saurav Panda, Mauro Andrisani, Massimo Del Fabbro, Gianluca Martino Tartaglia

**Affiliations:** ^1^ Department of Biomedical, Surgical and Dental Sciences, University of Milan, 20122, Milan, Italy, unimi.it; ^2^ Foundation IRCCS Ca’ Granda Hospital Maggiore Policlinic, 20122, Milan, Italy; ^3^ Department of Oral and Maxillofacial Surgery, Faculty of Dentistry, Istanbul Aydın University, Istanbul, Türkiye, aydin.edu.tr; ^4^ Private Clinic, Duccio di Buoninsegna Street 48, 50143, Florence, Italy; ^5^ Department of Periodontics, Institute of Dental Sciences, Siksha ’O’ Anusandhan University, 751002, Bhubaneswar, Odisha, India, ids.ac.in

**Keywords:** DC-TMD Questionnaire, diathermy, temporomandibular disorders, TMD-related pain, TMJ

## Abstract

**Background:**

The temporomandibular disorder (TMD) treatment modalities range from noninvasive to surgical treatments. Diathermy is one of the noninvasive medical treatment options in which controlled high‐frequency energy is applied to generate heat in body tissues to treat osteoarthritis and muscle pain. The aim of this pilot study was to evaluate diagnostic criteria for temporomandibular disorder (DC‐TMD) Questionnaire based results of diathermy stimulation rehabilitation in patients with temporomandibular joint (TMJ) problems with no internal dislocation of the disc.

**Methods:**

The DC‐TMD questionnaire documents were uploaded to a special question evaluation software, and 47 questions (grouped in 8 categories) were automatically chosen to assess TMD‐related pain, jaw mobility, and quality‐of‐life impact. All the patients received diathermy stimulations that were scheduled once a week for 5 weeks (*T*0: 1st diathermy simulation, *T*1: 2nd session, *T*2: 3rd session, *T*3: 4th session, *Tf*: 5th session). Pre and post‐treatment results from 47 questions were collected at each session, and outcomes were assessed among them, using *T*0 as a baseline to evaluate improvements.

**Results:**

10 patients (8 women and 2 men) aged between 22 and 50 years were included. There was a tendency for improvement for each category, and mean scores decreased over time. However, no significant difference was found in overtime for any category except the Mandibular Functional Limitation Scale (LM), which showed significant improvement, and patients reported fewer limitations.

**Conclusions:**

The outcomes obtained cannot be considered total recovery from problems, but according to the results, diathermy applications can be regarded as beneficial for improving TMJ‐related disorders, which can increase the quality of life (QoL) of such patients.

## 1. Introduction

Temporomandibular disorders (TMDs) were defined by the American Academy of Orofacial Pain (AAOP) as a common clinical problem of muscular and neuromuscular pathologies that affect the temporomandibular joint (TMJ) and the associated structures, causing tenderness in the orofacial region with the presence of TMJ sounds and restricted movements in the jaw during opening or closing of the mouth [1]. According to a study by Zielinski et al. [2], the prevalence of TMDs among Europeans was 29%, ~15% in adults, and 7% in adolescents. There are several ways to diagnose TMDs, including the diagnostic criteria for TMDs (DC‐TMDs), a widely used standardized system to analyze and classify TMDs according to a biopsychosocial model [3, 4]. In 1992, DC‐TMD set forth a dual‐axis diagnosis: Axis I dealt with the physical aspects of TMD, and Axis II evaluated psychosocial factors of pain [3]. Therefore, the questionnaire included a series of questions related to the frequency, intensity, and duration of pain and the impact caused by TMD on daily activities [5]. It also considers the individual’s emotional condition and the perception of symptoms expressed by the patient. Hence, it provides a comprehensive diagnosis of TMD and helps the clinician to plan proper treatment that is oriented toward the specific needs of each patient, especially concerning the interaction of physical and psychological components [1, 4, 6].

The chronic nature of TMD makes effective management difficult; thus, clinicians and researchers continue to seek alternative treatment modalities, one of which is the use of heat through diathermy [7–10]. Diathermy is a noninvasive option that can provide some comfort in less time when compared with occlusal splints, occlusal plates, and Botox injections [11–13]. Its applications are used with successful outcomes as treatments in physiotherapy to generate deep heating to relax muscles and joints, reduce inflammation, and improve blood circulation. The results of diathermy can be seen in 4–5 weeks, and it does not cause any discomfort to the patients as reported by other treatment modalities [14–16]. Various researchers reported successful outcomes. Pundkar et al. reported that ultrasound diathermy decreased the TMD symptoms and improved the movement of the jaws [17, 18]. Melis and his colleagues [18] have reported that shortwave diathermy provided symptom relief in patients with TMJ disorders, such as less pain, improved jaw mobility, and more relaxed mandibular elevator muscles.

Currently, there are limited reports in the literature, and there is no established standard treatment protocol for the applications of diathermy in TMD patients, which also investigates adverse effects such as damage or burns to tissues [19]. Variability in frequency, duration, and intensity during the treatments has made the comparison between studies quite difficult, hence limiting the conclusions for the best therapeutic option [20]. The use of DC‐TMD might provide a standard for outcome measurements and allow comparisons between studies in the future [4]. This research aimed to investigate whether diathermy stimulation is effective or not for the improvement of TMD‐related muscle pain and dysfunction in TMD patients with no internal dislocation of the disc, using the DC‐TMD Questionnaire. As an objective, the results might also fill the gaps in the literature concerning standardized treatment modalities and the short‐term durability of treatment effects.

## 2. Materials and Methods

This retrospective, nonrandomized case series aimed to evaluate the outcomes of the diathermy stimulation technique using the intraoral handpiece of the Velvet TMJ (Top Quality Medical, Castello, Italy) equipment using the questions that were chosen from DC‐TMD Questionnaire, which is special for TMD patients. The evaluation protocol used in this study was approved by the Ethics Committee of Area 2 Milano (Prot. No. 575‐2018, Date: July 17, 2018). The study followed the principles laid down in the Declaration of Helsinki on medical protocol and ethics. All participating patients had signed the informed consent before the procedure. The STROBE guidelines for observational studies were followed in this work.

The information to be evaluated was collected from the clinical records according to the inclusion criteria from the database of patients who had TMJ problems and who had received treatment at a Milan University clinic with Velvet TMJ between February 2022 and February 2024.

### 2.1. Inclusion Criteria

Axis I and II patients with TMJ problems/complaints but without any internal dislocation of the TMJ (or the situation was not clinically undetected and the problem was related to muscular problems); good hygienic conditions; able to understand the study requirements and compliance with the instructions and plan of appointments, as well as giving their approval to participate in the study through a written informed consent form before treatment; and patients that were otherwise healthy, based on their medical history and physical examination.

### 2.2. Exclusion Criteria

Oncologic patients; patients with a history of drug or alcohol abuse; patients suffering from psychosis and endocrine disorders; patients who were unable to maintain reasonable standards of home hygiene to follow the requirements of the study; patients who were pregnant or breastfeeding at the time of recruitment; and patients at a developmental age.

Currently, magnetic resonance imaging (MRI) is considered the gold standard for soft tissue imaging for visualizing joint effusion, disc position, shape, and integrity for TMJ. This study included TMD patients with no internal dislocation of the disc with soft tissue problems; the diagnosis was made by a single operator who has expertise on this topic (Andrea Gizdulich) by clinical examination verified with recent MRI images.

All the patients had bilateral TMJ pathology due to muscular problems, and each received a diathermy stimulation with a handpiece of the Velvet TMJ. No other adjunctive therapy was utilized.

### 2.3. Diathermy Stimulation Technique

A metal plate was used to direct the radio waves generated by the handpiece at a controlled frequency and direction. A conductive gel was used between the metal plate to reduce the impedance. All the patients received extra‐oral diathermy applications bilaterally over the skin, including cheek, mandibular angle, and TMJ region. The region of interest was prepared, making sure the patient’s skin is intact, clean, and dry before application. Fisiowarm Cream (by Golden Star Srl., Rome, Italy) was spread on the area to be treated to improve the contact and smoothness of the electrode. During treatment, it was continuously monitored to ensure the cream remained between the electrode and skin for optimal results. Subsequently, according to the instructions from the supplier and manufacturer, the treatment was performed. The handpiece of the Velvet TMJ equipment was used to stimulate only one side of the patient’s TMJ during each session. Each single‐polar capacitive and resistive session (1000 kHz) lasted a total of 40 min (20 + 20 min) at 7% power in “edema and tumefaction treatment” mode. These were standardized across all sessions and patients. All patients were treated by the same operator (Andrea Gizdulich), who performed the applications according to the parameters indicated by the supplier for the Velvet TMJ device with capacitive and resistive stimulation to stimulate tissues with high water content, such as muscle fibers, and with low water content, such as tendons and ligaments. Diathermy stimulations were applied once a week in five sessions (*T*0: baseline [1st diathermy simulation], *T*1: 2nd session, *T*2: 3rd session, *T*3: 4th session, and *Tf*: final diathermy simulation). All patients had received a total and complete cycle of 20 min of capacitive and 20 min of resistive applications once a week for 5 weeks of treatment. The treatment outcomes were assessed among them to observe the progress, and *T*0 was used as a baseline to evaluate improvements.

The protocol of this study followed the instructions recommended by the manufacturer. During the applications and in the post‐treatment period, no specific patient intolerance regarding the diathermy was experienced. According to the protocol, there was no specific therapy or medication following the diathermy applications. The subjects did not receive any kinesiotherapy or anti‐inflammatory medications, pain killers, or muscle relaxants. No therapeutic exercise was administered to the patients. As recommendations, the participants were instructed to avoid excessive hot/cold drinks/meals, hot showers, and heavy physical exercises for the following 24 h. The patients were recalled for routine follow‐ups every 6 months, and in case they had any problems/adverse effects/worsening of their situation after the treatments, they were told to inform the clinic immediately.

### 2.4. DC‐TMD Questionnaire

After treatment with diathermy, all TMD patients (with no internal dislocation of the disc) filled out 47 questions that were selected from the DC‐TMD questionnaire (documents are available at www.DC-tmdinternational.org) based on the 2 axes of observation independently and voluntarily during the visits [https://www.aignatologia.it/web/app/uploads/2020/04/DC-TMD-Italian-Assessment-Instruments_2017-02-10-2.pdf, Ohrbach R (editor) (2016). DC‐TMDs: Assessment Instruments. Ohrbach R, Knibbe W (2016). DC‐TMDs: Scoring Manual for Self‐Report Instruments. Ohrbach R, Gonzalez YM, List T, Michelotti A, Schiffman E (2014). DC‐TMDs: Clinical Examination Protocol]. The standard versions of the DC‐TMD Questionnaire documents are in English, and translated versions are available in several languages. The original questionnaires were uploaded to a special question evaluation software that uses a system of calculating the scores based on original assessment tools (which can be accessed at the website www.questionwriter.com). Considering the study’s retrospective design, in order to reduce bias, questions were chosen randomly by a specific software that uses artificial intelligence. A category name was attributed automatically for each question, and subsequently, they were organized into sections according to the international criteria. For each patient and each session, the questions were randomized by artificial intelligence to prevent the patients from identifying the tested topic group and to prevent the memorization of previous answers. The questionnaire results were administered with a homogeneous calculation system in each step and for each answer (Table 1). 26 was taken as the highest score, and percentages were calculated accordingly to evaluate results. All scores were calculated in absolute value (26 as the worst value and subsequently as a percentage change from the initial timing of the test (*T*0). The questions of the TMD care questionnaire that were utilized in this research can be seen in Appendix S1, Table SA1.

**Table 1:** Categories of health parameters extracted from the questionnaires.

ANX: Anxiety disorder score (this category of questions is based on the diagnoses of anxiety disorders in a general sense. Its value represented the degree of perception and psychological suffering and the degree of impact on social and relationship life. This indicator can be influenced by systemic factors and psychiatric diseases as an etiological factor of TMD, but is also useful for identifying psychological risk factors in patients subject to occasional stress).
PHQ: Depression disorder (this score is based on the degree of disability from a depressive disorder. The questions were extracted for mental health and hidden depression which are known as contributory causes of TMD).
DC: Chronic pain grading (a score on chronic pain reported in the last 30 days with a VAS scale of 0–10. Chronic pain analysis was used to visualize the intensity of pain, and the risk of chronic pain associated with some forms of TMD. Its value expresses both the subjective level of perception of pain and the possibility that the chronic pain itself is the cause of TMD).
LM: Mandibular Functional Limitation Scale (score on the functional limitation perceived by the patient to recognize the degree of limitation of the masticatory system. The scale showed the degree of limitation in carrying out the main daily activities. Mild or moderate chewing parafunctions).
Co: Bruxism and score on oral behavior associated with bruxism as perceived by the patient (involuntary parafunctions, such as bruxism or clenching, which can be occasional and spontaneous).
G1: Score relating to muscle pain present in the last 30 days (the TMJ joint is free from obstacles but there may be slight overload phenomena without anatomical damage. In the presence of limitations in mandibular movement or other daily activities of the mouth, involvement of the nearby pterygoid muscles is considered as muscular pain).
G2A: TMJ sounds (score on the noise of the temporomandibular joints).
G2B: TMJ blocking (score on the tendency to block the temporomandibular joints).

### 2.5. Evaluation Method for Statistics

Statistical analysis of data was carried out using the Graphpad software version 5.03. Pre vs. post‐treatment values were collected at five different timelines (*T*0: baseline [1st diathermy simulation], *T*1: 2nd session, T2: 3rd session, *T*3: 4th session, *Tf*: final diathermy session). The highest score of the index (*n* = 26) represented the worst situation, while zero represented the best value, with fewer complaints, problems, and TMJ function limitations. A decrease in the baseline assessment’s value was considered an improvement in the condition involving TMJ. The results were subsequently transformed into percentages for normalization, and comparison was done among different time frames. The pattern of scores % over time, mean value, and standard deviation (SD) of scores as percentages were assessed. *p*‐values ≤0.05 were accepted as being significant. The results were analyzed using a nonparametric approach using the Friedman test followed by the Dunn post hoc test.

## 3. Results

Ten patients (8 women and 2 men) aged between 22 and 50 years were selected according to the inclusion criteria. There was a tendency for improvement for each category, and mean scores decreased over time. No adverse events were seen in any of the patients.

**Figure 1:**
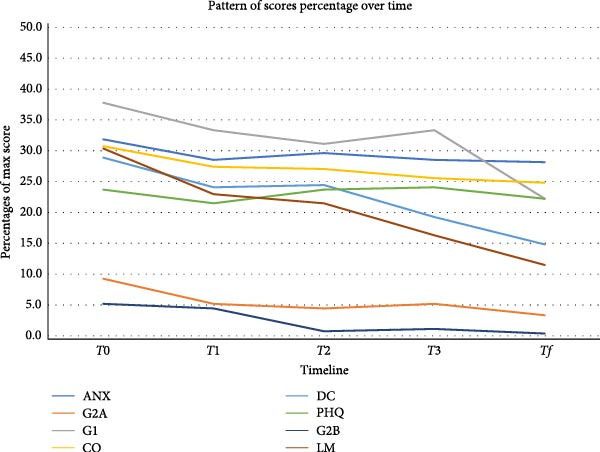
The graph shows the mean values (MVs) and standard deviations (SDs) for percentage scores of various health parameters related to temporomandibular disorder (TMD) across five‐time points during a 5‐week diathermy treatment. These parameters include anxiety (ANX), TMJ sounds (G2A), muscle pain (G1), oral behaviors (CO), chronic pain grading (DC), depression disorder (PHQ), TMJ blocking (G2B), and mandibular functional limitation (LM).

Figure 1 shows that parameters like anxiety (ANX), TMJ sounds (G2A), muscle pain (G1), chronic pain grading (DC), TMJ blocking (G2B), and mandibular functional limitation (LM) exhibited a downward trend, indicating an overall improvement in patient conditions. According to the outcomes, ANX showed a moderate decrease from T0 (31.85%) to Tf (28.15%), while G2A significantly declined from 9.26% to 3.33%, suggesting a positive impact of diathermy on joint sounds. Similarly, G1 and DC decreased steadily over time, with muscle pain showing a substantial reduction from 37.78% to 22.22%, indicating notable pain relief. CO and PHQ remained relatively stable, with minor fluctuations, indicating that these parameters might be less responsive to diathermy or influenced by other factors. G2B demonstrated a sharp decline, from 5.19% at T0 to 0.37% at Tf, suggesting significant improvement in joint mobility. The *p*‐values provided statistical insight into the significance of these changes. Among all parameters, only LM showed a statistically significant reduction (*p* = 0.0216), suggesting that diathermy notably improves jaw functionality. Other parameters, despite showing decreasing trends, do not reach statistical significance, which may be due to sample size limitations or variability in patient responses. LM followed a similar pattern, reducing from 30.37% to 11.48%, reflecting enhanced mandibular function. Table 2 shows the mean values and SD values of scores as percentages and *p*‐values.

**Table 2:** Mean values (MVs) and standard deviation (SD) values of scores as percentages.

Time	Health Parameters															
	ANX		G2A		G1		CO		DC		PHQ		G2B		LM	
	MV	SD	MV	SD	MV	SD	MV	SD	MV	SD	MV	SD	MV	SD	MV	SD
*T*0	31.85	18.33	9.26	9.44	37.78	14.05	30.74	13.97	28.89	23.86	23.70	12.49	5.19	10.06	30.37	20.57
*T*1	28.52	16.66	5.19	7.03	33.33	13.52	27.41	12.85	24.07	18.66	21.48	14.38	4.44	8.52	22.96	16.64
*T*2	29.63	16.47	4.44	4.88	31.11	14.42	27.04	14.19	24.44	21.25	23.70	16.49	0.74	2.34	21.48	16.27
*T*3	28.52	14.40	5.19	5.58	33.33	13.18	25.56	13.69	19.26	16.45	24.07	17.31	1.11	3.51	16.30	13.09
*Tf*	28.15	12.37	3.33	4.77	22.22	11.04	24.81	10.34	14.81	11.84	22.22	15.71	0.37	1.17	11.48	10.25
*p*‐Value	0.9686		9.1665		0.3731		0.7783		0.4736		0.999		0.2805		0.0216 ^∗^	

*Note*: ANX, anxiety disorder score; Co, bruxism and score on oral behavior associated with bruxism; DC, chronic pain grading; G1, score relating to muscle pain present in the last 30 days; G2A, TMJ sounds; G2B, TMJ blocking; LM, Mandibular Functional Limitation Scale; PHQ, depression disorder.

^∗^Statistical significant value.

## 4. Discussion

The first‐line treatment in TMD is usually a noninvasive option, mostly physical therapy, which focuses on exercises that improve jaw mobility, strengthen muscles, and reduce pain [3]. Occlusal Splints or bite guards are also common, which can stabilize the jaw, reduce muscular tension, and prevent bruxism [8]. Along with these therapies, analgesics, muscle relaxants, and anti‐inflammatory drugs are also mostly prescribed [3]. Although pharmacotherapy has symptomatic short‐term effects, it can also show side effects like gastrointestinal complications, and the addictive nature of opioids calls for prudent use [3, 8, 9]. When these conservative measures fail, minimally invasive procedures like arthrocentesis or intra‐articular injections with steroids can provide symptomatic temporary relief for the patients [5, 7, 8]. As a last option, surgery is taken into consideration for the most extreme scenarios of TMDs, like people who have structural abnormalities or degenerative joint diseases [9]. Surgical interventions can achieve long‐term symptom relief, but they often carry higher risks of infection and neural damage, hence reduced jaw functioning [9, 10].

Currently, as a noninvasive option, ultrasound, radiofrequency therapy, electrical stimulation, and diathermy are being used to reduce edema and improve joint functioning [11–16]. These methods are considered to provide mild pain relief and functional improvement, but generally, they are applied in combination with other conservative treatments like cognitive behavioral therapy [3, 4]. In this pilot study, the diathermy stimulation technique was used with radiofrequency of 500 KHz and 1 MHz. Diathermy is a form of endogenous thermotherapy based on the use of heat for therapeutic purposes, especially in the presence of painful and muscular conditions. It is a painless and noninvasive solution capable of treating the tissues of the stomatognathic system and TMDs [16]. It works on the principle of raising the temperature of tissues, hence improving blood flow, reducing inflammation, and reducing the stiffness of the muscles, thereby promoting healing [21]. In TMD, the therapy aims to decompress the joints, relax the muscles, and contribute to the repositioning of the jaw. However, in cases of improper applications, there is always a risk of tissue burn [16, 22]. Furthermore, diathermy has limited applications, hence it is contraindicated in patients with pacemakers, metal implants in the body, and abnormalities in the skin such as acne, infection, and tumor, which can hinder its wide usage [23].

Most studies on diathermy and ultrasound therapy are based on musculoskeletal disorders (MSDs), and there is a limited number of studies for treating TMD, which did not have very strong evidence‐based methodology [15, 16, 19, 21]. Recently, Talaat et al. [24] compared shortwave diathermy with ultrasound therapy and a combination of methocarbamol and acetylsalicylic acid. They found no significant difference between shortwave diathermy and ultrasound therapy; also, diathermy was superior to the medication combination for post‐treatment pain relief [24]. Besides, there is some proof of the short‐term effectiveness of diathermy for muscle relaxation and dissipating pain, but the long‐term impacts of it on TMJ functions and patient results remain unclear [25, 26]. These gaps highlight the need for new investigations to elucidate its sustained benefits and safety profile.

A recent systematic review and meta‐analysis conducted by Pollet J et al. [16] investigated the efficacy of electromagnetic diathermy, including shortwave, microwave, and capacitive resistive electric transfer, for MSDs. The review included 68 studies involving 4892 patients. Key outcomes assessed were pain, function, quality of life (QoL), and adverse effects, with studies covering a range of durations from post‐treatment assessments to long‐term follow‐ups. The conditions treated included osteoarthritis and low back pain. This review found evidence suggesting that diathermy could relieve pain, improve function, and enhance QoL, potentially due to increased blood flow, oxygenation, and possible activation of the central nervous system [16]. Other similar studies conducted by Adly et al. [21], Gray et al. [27], and Dhanasekaran et al. [28] evaluated the effectiveness of shortwave diathermy in managing TMJ disorders [21, 27, 28]. As a result, they found that short‐wave diathermy showed faster symptom relief when compared with other therapies [21, 27, 28]. Similarly, the results of this present study showed that muscle pain and chronic pain grading decreased steadily over time, with muscle pain showing a substantial reduction from 37.8% to 22.2%, indicating notable pain relief.

In another clinical research, Adly et al. [21] measured the treatment outcomes based on clicking reduction assessed via stethoscope evaluation. The clicking reduction was found to be more pronounced in the placebo group (94.8%) compared to the experimental group (52.4%) immediately post‐treatment. The study concluded that shortwave diathermy is effective for TMJ pain relief but less so for addressing joint clicking [21]. However, in the present study, G2A, declined critically from 9.26% to 3.33%, suggesting a positive impact of diathermy on TMJ sounds.

Furlan et al. [29] conducted an integrative review to evaluate the use of superficial heat therapy for managing TMD. The review focused on application techniques, durations, treated body areas, temperatures, frequencies, and the associated benefits of superficial heat therapy. The results highlighted that moist heat was the most commonly used technique, typically applied for ~20 min daily to the face and neck. Common methods included warm compresses, electric heating pads, and silica gel pads. Reported benefits included pain relief, reduced muscle tension, improved mandibular function, and increased mouth opening [29]. Despite these benefits, the studies showed significant variability in methodologies, and superficial heat was often combined with other treatments, such as occlusal splints, muscle stretching, and dietary modifications [29]. However, drawbacks included the complexity of the application, which affected adherence, potential symptom worsening in sensitive individuals, and limited effectiveness when compared to more advanced techniques like diathermy [29].

Currently, there is very limited literature with no clear and detailed protocols on diathermy applications in oral medicine [19, 27–32]. Although there are still a limited number of papers on TMD, diathermy applications were evaluated since 1950s. As Royer et al. [31] had investigated outcomes of diathermy for swelling and trismus at 100 W, 5 cm from the cheek, which was applied for 20 min. In a more recent study by Dhanasekaran et al. utilized pulsed shortwave therapy (pSWT) operated at 27.12 MHz was delivered with short pulses of a magnetic field at a frequency of 1000 pulses per second for five consecutive days and reported good pain relief without adverse effects in TMD patients [28]. Additionally, the scientific papers that are present show a variety of protocols for the management of different conditions (such as management of sinusitis, TMJ pain, intraoral mucosal wound healing, orthodontic tooth movement, trismus, etc.) [19, 27–32]. In a clinical research, Gray et al. [27] evaluated results for TMD pain dysfunction, but only the parameter described for the application of diathermy was a mild thermal setting. Similarly, Kalekar et al. [30] evaluated the effect of therapeutic ultrasound versus shortwave diathermy combined with suboccipital release and manual drainage techniques for chronic sinusitis, but the protocol for frequency, intensity was not clearly mentioned. The diathermy was applied in continuous mode with an intensity according to the tolerance of the patient for 10 min of treatment. Total duration of the treatment was 30 min/session for five consecutive days [30]. In a case report, Curilli et al. [32] utilized microwave diathermy in orthodontics, a single‐pole resistive session at 1000 kHz, set to 7% power in “fracture consolidation treatment” mode for 6 min, weekly for 3 weeks [32]. Due to the variety of protocols and situations reported in scientific publications, currently, there is no specific gold protocol confirmed scientifically for diathermy applications for TMD patients. And due to this reason, this present work simply relied on the instructions from the manufacturer, which were similar to the protocol explained by Curilli et al. that used diathermy for orthodontic purposes [32].

In the present work, all the patients received diathermy applications as single‐polar capacitive/resistive sessions once a week (1000 kHz) of 40 min at 7% power. These were standardized across all sessions and patients. A 5‐week period was chosen due to its ability to reduce pain, inflammation, and muscle tension by heating the soft tissues, which can be beneficial for the condition. This is the typical time frame for diathermy procedures, which removes abnormal cells, and was set as a standard protocol to address the TMD symptoms. The results of this pilot study showed a trend of improvement over time in all categories, with LM showing a statistically significant reduction (*p* = 0.0216), indicating better jaw mobility and fewer functional restrictions. Other parameters exhibited improvement trends but did not achieve statistical significance. This finding may suggest the potential effectiveness of diathermy treatment for improving the outcome of TMD management and support the reports in the literature [16, 19, 21, 22]. Furthermore, this study can be considered unique because it mainly focused on the diathermy results based on the satisfaction of patients. This integrated patient feedback can help optimize the diathermy protocols and explore its role in the therapy for TMD.

The major limitation of this study was the short‐term evaluation of diathermy applications with a very limited number of patients. The latter was due to the fact that, as a pilot study aim was to collect original data that can serve to plan further prospective studies with a more robust sample size. Another point is that the evaluation technique for the results is simply based on the statements and declarations from the patients, which were provided as answers to the selected questions from a specific questionnaire. Further limitations are based on the nature of this investigation, since this is a retrospective work, nonrandomized, not blinded, and there is no control group. Moreover, the nature of the intervention applied did not allow for the blinding of the clinician and the participanting patients. More clinical studies should be planned as prospective, double‐blinded, and randomized with control groups using different evaluation methods to obtain results that can produce a higher scientific impact in the literature.

Another limitation of this work is that the Question Writer software is no longer available. By using this software in the clinics, it was possible to evaluate treatment outcomes that were made automatically online. In this study, the questions that were chosen for assessment of TMD patients by the Question Writer software can be tracked easily in supporting files (Appendix S1, Table SA1), with outcomes reported in the results section. This study is a part of the continuing research on the evaluation of rehabilitation of TMJ disorders. In our clinic, further studies with larger sample sizes and longer follow‐up periods are planned and are still under investigation using a specific AI‐based webapp developed to confirm and assess the effectiveness of various treatment methods for patients with different TMJ problems.

## 5. Conclusions

This work aimed to investigate whether diathermy stimulation is effective for TMD‐related pain, jaw mobility, and quality‐of‐life impact. Currently, there are very limited reports and no established protocol for diathermy, so the results obtained can be considered preliminary research and can help fill the gaps in the literature concerning standardized treatment modalities and the short‐term durability of treatment effects. The results obtained cannot be considered as total healing or remission from symptoms or total recovery from TMD complaints, however, according to the opinion of the authors, diathermy applications can be considered beneficial for an improvement in TMJ limitations and pain which as a result can improve the QoL of TMD patients (especially for patients with no internal dislocation of the disc) in short term. Further studies and research are needed in the literature to justify diathermy parameters (such as frequency, intensity, and duration) with specific application protocols for TMD patients.

**Nomenclature**
AAOP: American Academy of Orofacial Pain
ANX: Anxiety disorder
Co: Oral behaviors
DC: Chronic pain grading
DC‐TMDs: Diagnostic criteria for TMDs
G1: Muscle pain
G2A: TMJ sounds
G2B: TMJ blocking
LM: Mandibular Functional Limitation Scale
MRI: Magnetic resonance imaging
PHQ: Depression disorder
QoL: Quality of life
SD: Standard deviation
TMDs: Temporomandibular disorders
TMJ: Temporomandibular Joint.

## Ethics Statement

The protocol for retrospective evaluation of treatment outcomes of TMJ Disorders was approved by the Ethics Committee of Area 2 Milano (Prot. Number 575‐2018, Date: July 17, 2018), and this study was a part of this continuing research on this topic. The study was conducted in accordance with the Declaration of Helsinki.

## Consent

Informed consent was obtained from all subjects involved in the study.

## Disclosure

All authors have read and agreed to the published version of the manuscript. This manuscript was presented as a poster at the National Congress “32th National Congress of the Board of University Professors of Dental Disciplines on 18–20 June 2025.” The link of this local congress with the mentioned clinicians as authors can be found below in the reference section [33].

## Conflicts of Interest

The authors declare no conflicts of interest.

## Author Contributions

Conceptualization, methodology, software: Andrea Gizdulich, Gianluca Martino Tartaglia, Massimo Del Fabbro, Funda Goker, Ishita Singhal, Saurav Panda, and Mauro Andrisani. Validation: Andrea Gizdulich, Gianluca Martino Tartaglia, Massimo Del Fabbro, Funda Goker, Ishita Singhal, and Saurav Panda. Formal analysis, data curation, writing – original draft preparation, visualization, supervision, project administration: Andrea Gizdulich, Gianluca Martino Tartaglia, Massimo Del Fabbro, Funda Goker, and Ishita Singhal. Investigation: Andrea Gizdulich, Gianluca Martino Tartaglia, Massimo Del Fabbro, and Funda Goker. Resources: Andrea Gizdulich, Gianluca Martino Tartaglia, Massimo Del Fabbro, Funda Goker, Ishita Singhal, Saurav Panda, and Mauro Andrisani. Funding acquisition: Andrea Gizdulich, Gianluca Martino Tartaglia, Massimo Del Fabbro, Funda Goker, and Ishita Singhal.

## Funding

This study was partially funded by the Italian Ministry of Health, Current research IRCCS.

## Supporting Information

Additional supporting information can be found online in the Supporting Information section.

## Supporting information


**Supporting Information** The questions of the TMD care questionnaire that were utilized in this research can be seen in Appendix S1, Table SA1.

## Data Availability

The data that support the findings of this study are available upon request from the corresponding author. The data are not publicly available due to privacy or ethical restrictions.
