# Supplementary material for: Temporomandibular Disorder Treatment With Diathermy Stimulation Technique: A Pilot Study
Source: Int J Dent. 2025 Dec 8;2025:5486917. doi: 10.1155/ijod/5486917 (PMC12752898; doi:10.1155/ijod/5486917)
Supplement: Supplementary file 1 — Supporting Information The questions of the TMD care questionnaire that were utilized in this research can be seen in Appendix S1, Table SA1. [file IJOD-2025-5486917-s001.docx]

Appendix A

**Table A1.** List and categories of questions that were drawn from the TMD care questionnaire

| **QUESTIONS** | **1** | **2** | **3** | **4** | **5** | **CATEGORY** |
| --- | --- | --- | --- | --- | --- | --- |
| In the last 2 weeks, how often have you gotten angry or had worries that you couldn’t control? | Never | Sometimes | More than half of the days | About every day | NA | ANX |
| In the last 2 weeks, how often have you had trouble relaxing? | Never | Sometimes | More than half of the days | About every day | NA | ANX |
| Over the past 2 weeks, how often have you had trouble concentrating? | Never | Sometimes | Often | Always | NA | ANX |
| In the last 2 weeks, how often have you had little or too much appetite? | Never | Sometimes | Often | Always | NA | ANX |
| In the last 2 weeks, how often do you find yourself worrying too much about different things? | Never | Sometimes | More than half of the days | About every day | NA | ANX |
| In the last 2 weeks, how often have you felt bored or irritable? | Never | Sometimes | More than half of the days | About every day | NA | ANX |
| In the last 2 weeks, how often have you felt nervous or anxious? | Never | Sometimes | More than half of the days | About every day | NA | ANX |
| In the last 2 weeks, how often have you felt afraid about what might happen? | Never | Sometimes | More than half of the days | About every day | NA | ANX |
| In the last 2 weeks, how often have you found yourself talking or moving so slowly that other people noticed? | Never | Sometimes | Often | Always | NA | PHQ |
| Over the past two weeks, have you felt tired and low on energy? | Never | Sometimes | Often | Always | NA | PHQ |
| In the last 2 weeks, how often did you have low selfNAesteem or feel like a failure? | Never | Sometimes | Often | Always | NA | PHQ |
| In the last 2 weeks, how often have you been so nervous that you can't sit still? | Never | Sometimes | More than half of the days | About every day | NA | PHQ |
| In the last 2 weeks, how often have you had trouble staying asleep or sleeping too much? | Never | Sometimes | Often | Always | NA | PHQ |
| In the last two weeks, how many times have you been bothered by the thought that it would be better to be dead or to be hurt in some way? | Never | Sometimes | Often | Always | NA | PHQ |
| In the last 2 weeks, how often have you felt little interest or pleasure in doing things? | Never | Sometimes | More than half of the days | About every day | NA | PHQ |
| In the last 2 weeks, how often have you felt down, depressed, or hopeless? | Never | Sometimes | More than half of the days | About every day | NA | PHQ |
| How often do you play musical instruments that require the use of your mouth? | Never | Sometimes | Often | Always | NA | CO |
| How often do you chew gum? | Never | Sometimes | Often | Always | NA | CO |
| How often do you knock, press, or hold your teeth together in addition to chewing? | Never | Sometimes | Often | Always | NA | CO |
| How often do you press your tongue to your teeth or put your tongue between your teeth? | Never | Sometimes | Often | Always | NA | CO |
| How often do you clench or grind your teeth when you are half asleep or during the day? | Never | Sometimes | Often | Always | NA | CO |
| How often do you sleep in a position that compresses your jaw (e.g., on your side or your stomach)? | Never | 1NA2 nights/month | 1NA3 nights/week | 4NA7 nights/week | NA | CO |
| How often do you bite your tongue or play with your tongue or cheeks? | Never | Sometimes | Often | Always | NA | CO |
| How often do you bite objects like pens, pencils, and nails? | Never | Sometimes | Often | Always | NA | CO |
| How often do you bring your hand to your jaw so that it is held in a rigid position? | Never | Sometimes | Often | Always | NA | CO |
| In the last 30 days, how would you rate your facial pain as the worst? Use a 0NA10 scale, where 0 is "no pain" and 10 is "the pain is as bad as it gets". | 0NA10 | | | | | DC |
| In the past 30 days, how much has facial pain interfered with your daily activities? Use a 0NA10 scale, where 0 is "no interference" and 10 is "unable to carry on activities" | 0NA10 | | | | | DC |
| How do you rate your facial pain right now? Use a 0NA10 scale, where 0 is "no pain" and 10 is " the pain is as bad as it gets". | 0NA10 | | | | | DC |
| In the past 30 days, how much has facial pain interfered with your family and social activities? Use a 0NA10 scale, where 0 is "no interference" and 10 is "unable to carry on activities" | 0NA10 | | | | | DC |
| Please indicate your level of limitation in opening your mouth wide enough to eat or drink during the last 30 days. Please mark 10 if the activity was completely avoided. | 0NA10 | | | | | LM |
| Please indicate your level of restriction for chewing soft food during the last 30 days. Please, if the activity was completely avoided mark 10. | 0NA10 | | | | | LM |
| Please indicate your level of restriction in swallowing for the last 30 days, please mark 10 if the activity has been completely avoided. | 0NA10 | | | | | LM |
| Please indicate your level of restriction for chewing hard food during the last 30 days, please, if the activity has been completely avoided mark 10. | 0NA10 | | | | | LM |
| In the last 30 days, have you had headaches that include the temple area? | Never | A single episode | Often, about 1 day/week | Always, a few days a month without pain | NA | G1 |
| In the last 30 days, have you had pain when chewing hard or challenging foods? | Never | Sometimes, but I can finish chewing | Often, I need to be careful when chewing | Always, I have to avoid foods that are too demanding | NA | G1 |
| In the last 30 days, have you had pain when opening your mouth or moving your jaw forward or sideways? | Never | Sometimes | Often | Always | NA | G1 |
| In the last 30 days, have you had pain or discomfort in your mouth upon waking up? | Never | Sometimes | Often | Always | NA | G1 |
| In the last 30 days, how long have you had pain or discomfort in your jaw, temples, or elsewhere? | No pain or suffering | The pain comes and goes | The pain is always present |  | NA | G1 |
| In the past 30 days, have certain activities such as kissing, talking, or yawning increased your pain or discomfort? | Never | Sometimes | Often | Always | NA | G1 |
| In the last 30 days, when you open your mouth wide, have you experienced loud noises or clicking sounds, or has your mouth ever felt stuck so you couldn’t close it completely? | No, never | Sometimes, just a big fool but without pain or limitations | Often a big crash with pain and limitations that slowly resolve | All the time, my jaw locks and I have to reposition it by hand | NA | G2A |
| In the last 30 days, have you experienced any clicking or popping in your jaw joints when you move or use your mouth? | No, never | Sometimes but not always | Often | Always | NA | G2A |
| In the last 30 days, has your jaw ever locked so that you couldn’t open it fully, even for a moment, and then unlocked so that it came back free? | Never blocked | Yes, with spontaneous unlocks | Yes, it unlocks with the help of my hands | No, never unlocked again | NA | G2B |
| Has your jaw ever been locked or stuck so much that it interfered with your ability to eat? | Never | Sometimes | Often | Always | NA | G2B |
| Have you ever had your jaw lock or hold, even for a moment, so much so that you couldn’t open it fully? | Never | Sometimes | Often, also with joint pain | Always, I’m stuck right now too | NA | G2B |
| Observe your mouth opening and closing movement by looking at yourself in the mirror. Indicate the mouthNAopening path that best matches your natural movement. | Straight and linear movement | Slight lateral skids but substantially linear | Deviated to one side and then returned aligned to maximum opening | Permanently deviated to one side | NA | G2B |
| Measure your mouthNAopening ability using your fingers as a reference as shown in the figure. | 5 fingers | 4 fingers | 3 fingers | 2 fingers | 1 finger | G2B |

**NA:** Not Applicable
